# Substance use service availability in HIV treatment programs: Data from the global IeDEA consortium, 2014-2015 and 2017

**DOI:** 10.1371/journal.pone.0237772

**Published:** 2020-08-27

**Authors:** Angela M. Parcesepe, Kathryn Lancaster, E. Jennifer Edelman, Raquel DeBoni, Jeremy Ross, Lukoye Atwoli, Mpho Tlali, Keri Althoff, Judicaël Tine, Stephany N. Duda, C. William Wester, Denis Nash, for the IeDEA Consortium

**Affiliations:** 1 Department of Maternal and Child Health, Gillings School of Global Public Health, University of North Carolina at Chapel Hill, Chapel Hill, North Carolina, United States of America; 2 Carolina Population Center, University of North Carolina at Chapel Hill, Chapel Hill, North Carolina, United States of America; 3 Department of Epidemiology, The Ohio State University, Columbus, Ohio, United States of America; 4 Yale School of Medicine, New Haven, Connecticut, United States of America; 5 Yale School of Public Health, New Haven, Connecticut, United States of America; 6 National Institute of Infectology, Evandro Chagas, Fiocruz, Brazil; 7 TREAT Asia/amfAR, The Foundation for AIDS Research, Bangkok, Thailand; 8 Department of Mental Health, Moi University School of Medicine, Eldoret, Kenya; 9 Centre for Infectious Disease Epidemiology and Research, School of Public Health and Family Medicine, University of Cape Town, Cape Town, South Africa; 10 Department of Epidemiology, Johns Hopkins Bloomberg School of Public Health, Baltimore, Maryland, United States of America; 11 Maladies Infectieuses du Centre Hospitalier, National Universitaire de FANN, Dakar, Senegal; 12 Department of Biomedical Informatics, Vanderbilt University Medical Center, Nashville, Tennessee, United States of America; 13 Division of Infectious Diseases, Department of Medicine, Vanderbilt University Medical Center, Nashville, Tennessee, United States of America; 14 Vanderbilt Institute for Global Health (VIGH), Nashville, Tennessee, United States of America; 15 Institute for Implementation Science in Population Health, City University of New York, New York, New York, United States of America; 16 Department of Epidemiology and Biostatistics, City University of New York, New York, New York, United States of America; University of the Witwatersrand, SOUTH AFRICA

## Abstract

**Background:**

Substance use is common among people living with HIV and has been associated with suboptimal HIV treatment outcomes. Integrating substance use services into HIV care is a promising strategy to improve patient outcomes.

**Methods:**

We report on substance use education, screening, and referral practices from two surveys of HIV care and treatment sites participating in the International epidemiology Databases to Evaluate AIDS (IeDEA) consortium. HIV care and treatment sites participating in IeDEA are primarily public-sector health facilities and include both academic and community-based hospitals and health facilities. A total of 286 sites in 45 countries participated in the 2014–2015 survey and 237 sites in 44 countries participated in the 2017 survey. We compared changes over time for 147 sites that participated in both surveys.

**Results:**

In 2014–2015, most sites (75%) reported providing substance use-related education on-site (i.e., at the HIV clinic or the same health facility). Approximately half reported on-site screening for substance use (52%) or referrals for substance use treatment (51%). In 2017, the proportion of sites providing on-site substance use-related education, screening, or referrals increased by 9%, 16%, and 8%, respectively. In 2017, on-site substance use screening and referral were most commonly reported at sites serving only adults (compared to only children/adolescents or adults and children/adolescents; screening: 86%, 37%, and 59%, respectively; referral: 76%, 47%, and 46%, respectively) and at sites in high-income countries (compared to upper middle income, lower middle income or low-income countries; screening: 89%, 76%, 68%, and 45%, respectively; referral: 82%, 71%, 57%, and 34%, respectively).

**Conclusion:**

Although there have been increases in the proportion of sites reporting substance use education, screening, and referral services across IeDEA sites, gaps persist in the integration of substance use services into HIV care, particularly in relation to screening and referral practices, with reduced availability for children/adolescents and those receiving care within resource-constrained settings.

## Introduction

Substance use, in the form of the consumption of alcohol, tobacco, and other injection and non-injection drugs, is common among people living with HIV (PLWH) and has been associated with suboptimal HIV treatment access and outcomes, including higher lost to follow-up rates and mortality [1, 2]. In many global settings, alcohol is the most commonly used substance among PLWH. Severity of alcohol use has been associated with suboptimal HIV outcomes including late ART initiation, poor adherence to combination antiretroviral therapy (ART), lack of sustained viral suppression, and all-cause mortality [3–10]. Tobacco use has been reported at higher rates among PLWH compared to the general population and has been associated with poor health outcomes, including increased AIDS- and non-AIDS-related mortality [11–15]. It has been estimated that approximately 2.8 million people who inject drugs (PWID) are living with HIV globally [16]. PWID are approximately 28 times more likely to be living with HIV compared to the general population [17]. Non-injection drug use, including use of cocaine, opioids, and methamphetamine, has also been reported by PLWH [18–20]. Injection and non-injection drug use have been associated with suboptimal HIV treatment outcomes throughout the HIV care continuum, including poor engagement into care, delayed HIV diagnosis, and suboptimal ART adherence [21–29].

Evidence-based approaches to screen and treat substance use are associated with reduced substance use and improved health outcomes [30–32]. Substance use-related harm reduction education has been associated with reduced risk behaviors and positive health outcomes [33]. However, the overwhelming majority of PLWH with a substance use disorder do not receive such services [34]. The 2016 UN Political Declaration on HIV and AIDS called for more integrated service delivery for PLWH, including integration of substance use and HIV prevention and treatment services. Similarly, the UNAIDS 2016–2021 Strategy ‘On the Fast-Track to end AIDS’ recommends integration of comprehensive substance use and HIV prevention and treatment services for PWID. Integrating substance use services into HIV care is a promising strategy to improve both substance use and HIV-related care cascade outcomes and advance attainment of the UNAIDS 95-95-95 goals [35]. However, little is known about the availability or changes in availability over time of substance use services across HIV clinics globally. Greater understanding of the extent to which substance use-related services have been integrated into HIV care settings can inform the development, implementation, and evaluation of care integration strategies across geographic settings. In addition, such data are critically needed to inform mathematical models focused on the impact of integrated substance use and HIV care strategies.

In this paper we assess the availability and changes in availability of substance use-related education, screening and referral to treatment at HIV treatment centers participating in the International epidemiology Databases to Evaluate AIDS (IeDEA) consortium from 2014 to 2017 and identify research priorities related to the integration of substance use and HIV treatment services.

## Methods

The IeDEA consortium is a global research consortium established in 2006 of HIV treatment sites in seven geographic regions: Central Africa; East Africa; South Africa; West Africa; the Asia-Pacific; the Caribbean, Central, and South America (CCASAnet); and North America (NA-ACCORD) [36–39]. IeDEA is funded by the U.S. National Institutes of Health to collect globally diverse clinical observational HIV treatment data from PLWH. HIV care and treatment sites participating in IeDEA are primarily public-sector health facilities and are comprised of both academic and community-based hospitals and health centers [39, 40]. Because IeDEA sites have the capacity to routinely contribute electronic data, these sites may function at a higher level than HIV treatment sites not participating in IeDEA. While not representative of HIV treatment sites not participating in IeDEA, IeDEA HIV treatment sites have provided HIV care to nearly two million PLWH across the globe and serve as a major source of HIV care across global regions. Given the paucity of data regarding mental health and substance use care integration at HIV treatment centers globally, greater understanding of service provision at these sites can provide an important window into the status of care integration at relatively well-resourced HIV care and treatment sites across the globe.

IeDEA site assessment surveys are conducted approximately every two years. To be eligible to participate, sites must be active HIV treatment clinics and contribute data to IeDEA at the time of the survey. Research-only, non-clinical sites that do not contribute data to IeDEA are excluded. For the current analyses, we compared data from the two most recent site surveys. Data were collected between September 2014 and January 2015 and between June and December 2017. Both surveys collected information on characteristics of participating IeDEA sites including facility characteristics (e.g., location, level of care) and HIV treatment services.

In addition, sites were asked if they provided the following services to enrolled HIV patients:

- any education on high-risk substance use behaviors and harm reduction practices;
- any screening for drug or alcohol use; and
- any referral for substance use treatment.

Availability of substance use-related education, screening, and referral were categorized as occurring either on-site or off-site. On-site provision of substance use-related education, screening, or referral was defined as providing that service at the HIV clinic or the same health facility. Off-site provision of substance-use education, screening, or referral was defined as providing that service at another health facility or organization. The survey asked broadly about whether any substance use-related education, screening, or referral services were provided to HIV patients at each site. Thus, education-related services likely included a range of activities including one-on-one education and counseling as well as group-based educational programming. Similarly, screening services likely included a range of activities including the use of validated screening tools, assessment of biomarkers, and clinical inquiry.

English and French versions of the surveys were available online and in paper form. The online version was implemented using REDCap, a secure web-based data collection application hosted at Vanderbilt University [41]. Sites were instructed that surveys should be completed by individuals knowledgeable about clinic capacity and clinical services offered. The 2014–2015 site assessment was piloted at six sites from different regions and the 2017 site assessment was piloted at seven sites. Respondents shared feedback on survey content, response options, presentation, implementation in REDCap, and time to complete the survey. Minor changes were made to improve question clarity and the data entry interface based on feedback. IeDEA site assessments have been reviewed by the Vanderbilt University Human Research Protection Program Health Sciences Committee and received a non-human subjects determination.

Descriptive statistics summarized the prevalence of any substance use-related education, any screening, and any referral to treatment at IeDEA clinics in 2014–2015 and 2017. Analyses between site-level characteristics and substance use-related education, screening, and referral were conducted using chi-squared or Fisher’s exact tests, as appropriate. Income designation of the country in which the site was located was based on World Bank Income Designation of the country (as of July 2017). For sites that participated in both site assessments, McNemar’s tests were used to compare reported availability of substance use-related education, screening, and referral to treatment. A p-value of <0.05 guided interpretation of statistical significance. Analyses were performed using SAS Version 9.4.

## Results

**Fig 1:**
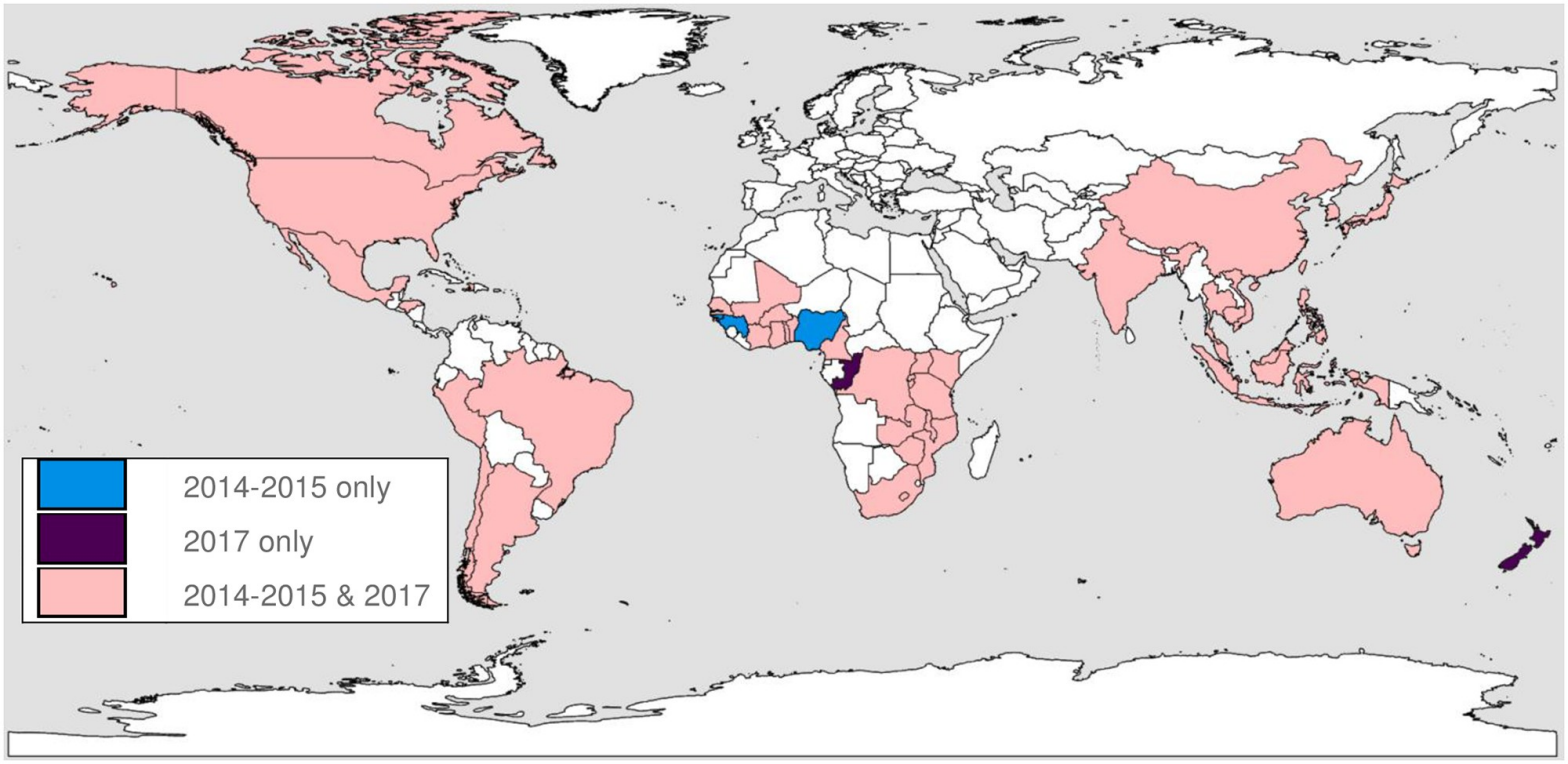
Geographic distribution of the HIV treatment sites from the IeDEA consortium participating in the 2014–2015 and 2017 global site surveys*. *Image created by the authors in ESRI’s ArcGIS 10.1; no copyrighted material was used [69, 70].

Among the 287 sites that were eligible to participate in the 2014–2015 site assessment and completed the survey, substance use-related data were available from 286 (99.6%) sites in 45 countries (Fig 1). Among the 255 sites that were eligible to participate in the 2017 survey, 237 (92.9%) sites from 44 countries completed the survey. In both surveys, the majority of sites were in urban areas (60% in 2014–2015; 75% in 2017) and were publicly funded (88% in 2014–2015; 85% in 2017). In both surveys, approximately half of sites provided care to both adults and children or adolescents (49% in 2014–2015; 46% in 2017). Approximately 40% of sites provided care to only adults (40% in 2014–2015; 42% in 2017) across surveys.

### Substance use-related services

In 2014–2015, most sites (75%, Range of percents Across IeDEA Regions [RAR] 42–100%) reported providing education to patients on substance use behaviors or harm reduction on-site (i.e., at the HIV clinic or the same health facility) and half reported on-site substance use screening (52%, RAR 6–89%) or providing referrals for substance use treatment (51%, RAR 0–91%) (Table 1). Forty-one percent of sites surveyed reported that substance use-related education, screening, and referral to treatment were all available on-site. Substance use-related education was reported as unavailable (either on-site or off-site) in 22% (RAR 0–51%) of sites. Substance use screening was reported as unavailable in 39% (RAR 0–71%) of sites, and referrals for substance use treatment were unavailable in 34% (RAR 0–76%) of sites. Twenty percent of sites reported that education, screening, and referral were all unavailable on-site.

**Table 1:** Substance use-related education, screening, and referral to treatment in HIV treatment programs within the IeDEA consortium, 2014–2015 and 2017.

	Site assessment, 2014–2015 n = 286		Site assessment, 2017” n = 237	
	Sites n (%)	RAR*%	Sites n (%)	RAR*%
**Education on high-risk substance use behaviors and harm reduction practices**				
On-site	215 (75%)	42–100%	200 (84%)	68–93%
Off-site	8 (3%)	0–8%	9 (4%)	0–14%
Not available	63 (22%)	0–51%	28 (12%)	5–26%
**Screening for drug or alcohol use**				
On-site	148 (52%)	6–89%	160 (68%)	29–90%
Off-site	27 (9%)	6–24%	15 (6%)	0–21%
Not available	111 (39%)	0–71%	62 (26%)	5–50%
**Referral for substance use treatment**				
On-site	146 (51%)	0–91%	139 (59%)	29–81%
Off-site	43 (15%)	9–24%	40 (17%)	11–50%
Not available	97 (34%)	0–76%	58 (24%)	2–50%
**Education, screening and referral all available on-site**	118 (41%)	0–84%	115 (49%)	14–71%

*RAR = Range of percentages across IeDEA regions

In 2017, 84% (RAR 68–93%) of sites reported providing on-site education, 68% (RAR 29–90%) reported on-site substance use screening, and 59% (RAR 29–81%) reported providing referrals for substance use treatment. Approximately half (49%) of sites reported that on-site substance use-related education, screening, and referral to treatment were all available on-site. Substance use-related education was reported to be unavailable (either on-site or off-site) in 12% (RAR 5–26%) of sites. Substance use screening was reported to be unavailable in 26% (RAR 5–50%) of sites, and referrals for substance use treatment were unavailable in 24% (RAR 2–50%) of sites. Eleven percent of sites surveyed in 2017 reported that education, screening, and referral were all unavailable on-site.

### Changes in on-site availability of substance use-related services from 2014–2015 to 2017

Among sites with data from both surveys (n = 147), the proportion of sites reporting on-site availability of substance use-related education, screening, or referral to treatment was compared between site assessments in 2014–2015 and 2017. Approximately 80% of sites reported on-site substance use-related education at both time points (83% in 2014–2015; 87% in 2017). There was a significant increase in the proportion of sites reporting on-site screening for substance use from 61% of sites in 2014–2015 to 74% of sites in 2017 (p<0.01) (Table 2). The proportion of sites that reported on-site availability of referral to substance use treatment increased from 57% in 2014–2015 to 66% in 2017 (p = 0.07).

**Table 2:** Availability of on-site substance use education, screening, and referral at HIV treatment programs participating in the 2014–2015 and 2017 surveys, IeDEA consortium (n = 147).

	Site assessment, 2014–2015 n (%)	Site assessment, 2017 n (%)	p-value
On-site education on high-risk substance use behaviors and harm reduction practices	122 (83)	128 (87)	0.26
On-site screening for drug or alcohol use	90 (61)	109 (74)	<0.01
On-site referral for substance use treatment	84 (57)	97 (66)	0.07
On-site education, screening and referral	73 (50)	84 (57)	0.12

**Fig 2:**
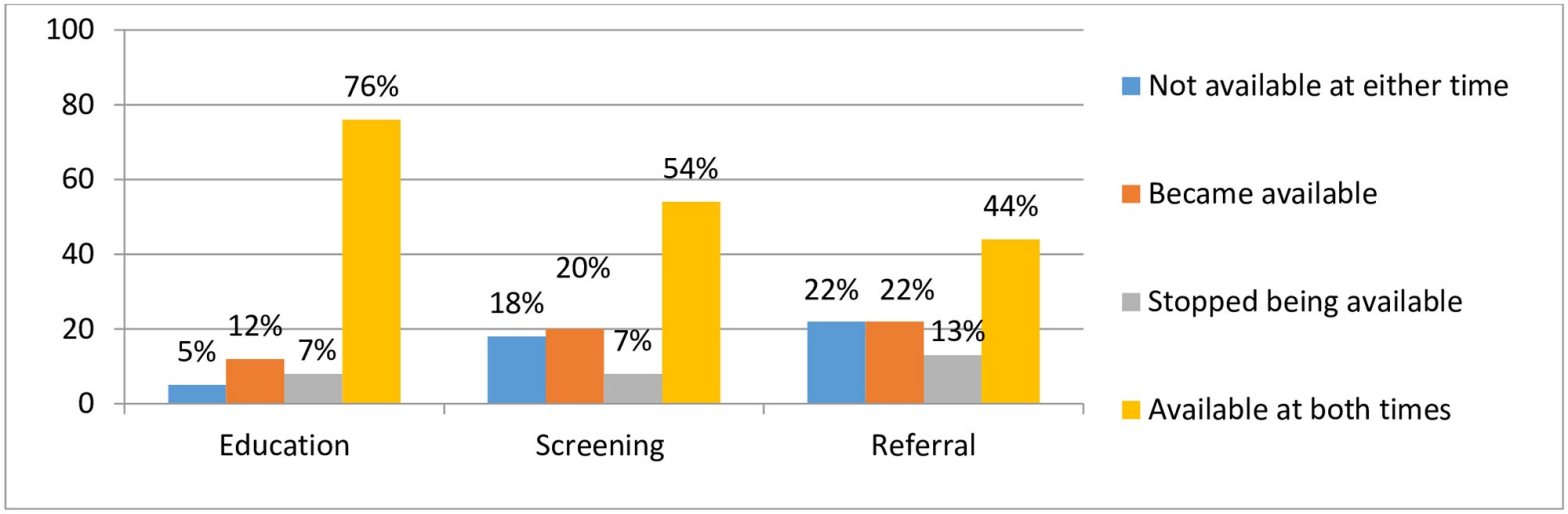
Changes in availability of on-site substance use-related education, screening, and referral at HIV treatment programs participating in the 2014–2015 and 2017 surveys, IeDEA consortium (n = 147).

Among sites with data from both surveys, substance use-related education was reported to be available on-site at both time points at 76% of sites and unavailable at both time points in 5% of sites (Fig 2). Twelve percent of sites reported that on-site substance use-related education became available at their site between 2014–2015 and 2017 and 7% reported that such education stopped being available during this timeframe (i.e., reported available in 2014–2015 and unavailable in 2017). On-site substance use screening was available at both time points at 54% of sites and unavailable at both time points at 18% of sites. Twenty percent of sites reported that on-site substance use screening became available during this period and 7% reported that screening stopped being available. On-site substance use referral was reported to be available at both time points in 44% of sites and unavailable at both time points in 22% of sites. Twenty-two percent of sites reported that substance use referrals became available during this timeframe and 13% reported that such referrals stopped being available.

### Service discontinuation between 2014 and 2017

A minority of sites reported having discontinued on-site substance use-related education (n = 11; 7%), screening (n = 11; 7%), or referrals (n = 19; 13%) between 2014–2015 and 2017. Sites that discontinued substance use-related education were distributed across IeDEA regions, facility levels (3 sites were in primary health centers and 8 were in regional, provincial, and teaching hospitals), types of patient populations served (4 sites served only adults, 3 served both adults and children or adolescents, and 4 served only children or adolescents), and were located in both urban and rural areas. All sites that discontinued on-site substance use-related education were publicly funded. Sites that reported having discontinued on-site substance use screening represented 6 IeDEA regions (all regions except Central Africa), rural and urban sites, various levels of health care facilities, and all types of patient populations served. Most (n = 10) sites that discontinued substance use screening were publicly funded. Similarly, sites that reported having discontinued substance use referrals were distributed across 6 IeDEA regions, (all regions except Central Africa) and served a variety of patient populations. Most sites that reported having discontinued on-site substance use referrals were regional, provincial, or teaching hospitals (n = 12), in urban areas (n = 12), and were publicly funded (n = 16).

### Site characteristics and availability of on-site substance use-related education, screening, and referrals, 2017

Overall, reported availability of on-site substance use-related education was common among sites surveyed. Availability of on-site substance use-related education was significantly associated with level of care of the health facility and with the geographic region in which the health facility was located (Table 3). On-site substance use-related education was most commonly reported by sites located in district hospitals and least commonly reported by sites located in regional, provincial, or teaching hospitals. These results should be interpreted with caution, however, as information on level of care of the health facility was missing for 11% of sites surveyed. Approximately 90% of sites located in North America, Asia-Pacific, and East Africa reported on-site substance use-related education compared to 68% of sites located in Southern Africa.

**Table 3:** Site-level characteristics and availability of on-site substance use-related education, screening, and referral at 237 HIV treatment sites within the IeDEA consortium in 2017^a^.

Characteristic	Total n	Education n (%)	Screening n (%)	Referral n (%)	Education, Screening, and Referral n (%)
**Setting**					
Urban/Mostly urban	157	135 (86)	117 (75)	102 (65)	86 (55)
Rural/Mostly rural	52	42 (81)	35 (67)	31 (60)	26 (50)
Unknown/Missing	28				
**Level of care**					
Primary (health center)	101	**90 (89)**	78 (77)	66 (65)	58 (57)
District hospital	17	**16 (94)**	14 (82)	10 (59)	8 (47)
Regional, provincial, or teaching hospital	92	**71 (77)**	60 (65)	56 (61)	45 (49)
Unknown/Missing	27				
**Patients treated at HIV program**					
Adults only	99	87 (88)	**85 (86)**	**75 (76)**	**65 (66)**
Children/adolescents only	30	21 (70)	**11 (37)**	**14 (47)**	**9 (30)**
Adults and children/adolescents	108	92 (85)	**64 (59)**	**50 (46)**	**41 (38)**
**Sector**					
Public	202	168 (83)	132 (65)	119 (59)	97 (48)
Private	35	32 (91)	28 (80)	20 (57)	18 (51)
**IeDEA region**					
Asia-Pacific	53	**47 (89)**	**42 (79)**	**41 (77)**	**35 (66)**
Caribbean, Central and South America	14	**11 (79)**	**9 (64)**	**6 (42)**	**5 (36)**
Central Africa	19	**15 (79)**	**10 (53)**	**8 (42)**	**7 (37)**
East Africa	58	**54 (93)**	**36 (62)**	**29 (50)**	**23 (40)**
North America	41	**37 (90)**	**37 (90)**	**33 (80)**	**29 (71)**
Southern Africa	38	**26 (68)**	**22 (58)**	**18 (47)**	**14 (37)**
West Africa	14	**10 (71)**	**4 (29)**	**4 (29)**	**2 (14)**
**Income level of country**^b^					
Low income	74	61 (82)	**33 (45)**	**25 (34)**	**18 (24)**
Lower middle income	60	51 (85)	**41 (68)**	**34 (57)**	**29 (48)**
Upper middle income	38	29 (76)	**29 (76)**	**27 (71)**	**22 (58)**
High income	65	60 (92)	**58 (89)**	**53 (82)**	**46 (71)**

^a^Differences that were significant at p <0.05 level of significance are highlighted in bold.

^b^Based on 2017 World Bank Income Designation of country in which site was located

Percentages were computed using the number of sites with a non-missing value.

On-site substance use screening was least commonly reported at sites serving only children or adolescents and most commonly reported at sites serving only adults (37% and 86%, respectively). On-site substance use screening was also least commonly reported at sites located in low-income countries as compared to sites in middle- or high-income countries. On-site screening was significantly associated with geographic region, with North American sites most commonly reporting screening and West African sites least commonly reporting screening.

On-site referral to substance use treatment was most commonly reported by sites that serve adults only as compared to sites that serve children or adolescents only or both adults and children or adolescents. On-site referral to substance use treatment was least commonly reported in sites located in low-income countries and most commonly reported in sites located in high-income countries. The majority of sites located in North America (80%) reported on-site referral to substance use treatment compared to 29% of sites located in West Africa.

## Discussion

Substantial gaps persist in the integration of substance use services into HIV care settings, particularly in relation to screening and referral to treatment, with reduced availability seen for younger patients (i.e., children and adolescents) and persons receiving HIV care within resource-constrained settings. Future context-specific research should investigate potentially modifiable, multilevel barriers and facilitators to the integration of substance use-related screening and referral, including barriers and facilitators at patient, provider, health facility, and policy levels. For example, U.S.-based research has demonstrated that the clinical care system for PLWH has not viewed substance use as a top priority and that U.S.-based HIV primary care providers have had limited knowledge and training to address alcohol use disorder [42, 43]. In addition, efforts to integrate substance use screening and referral into HIV care may be hampered by limited availability of validated, culturally adapted substance use screening tools and few linkages to substance use treatment, particularly in resource-constrained settings. Acceptable, feasible, and sustainable integration strategies that address these and other identified barriers and facilitators should be developed, implemented, and evaluated.

Sites participating in IeDEA change over time, creating a challenge to conducting longitudinal research in real-world service settings. Analysis of the clinics for which 2014–2015 and 2017 data were available found a statistically significant increase in reported availability of on-site substance use screening between 2014–2015 and 2017. While this increase is encouraging, data on factors associated with this increase are limited, and the site surveys did not explore details on how screening was conducted, the quality of screening, the specific substances for which screening was conducted, or who conducted screening. Future assessments of substance use service integration should include more nuanced, culturally-specific questions capable of assessing such aspects of screening and treatment services. A literature review examining substance use screening approaches in HIV care settings found that the use of validated substance use screening tools was limited and providers’ reliance on self-reported substance use was common [18]. Underreporting of substance use is common among PLWH due to stigma, medical mistrust, and concern about criminal justice implications of disclosure [44, 45]. HIV service settings that conduct substance use screening should use validated screening tools to identify substance use and guide treatment or referral decisions. In addition, where feasible, clinics should consider implementing web- or computer-based substance use screening protocols, as such methods may reduce underreporting and provider burden and increase screening and detection [18, 46].

A minority of clinics that reported availability of substance use-related education, screening, or referral in 2014–2015 reported that these services were no longer available in 2017. It is unclear what led to the discontinuation of these services. Substance use service discontinuation was reported by clinics across all IeDEA regions, patient populations, and by clinics in rural and urban areas. On-site referral to substance abuse treatment was the service most commonly reported to have been discontinued between 2014–2015 and 2017. Such findings highlight the need for longitudinal studies of service integration to better understand the feasibility and sustainability of service integration strategies. Little is known about factors associated with discontinuation of service integration. Research with clinics that implemented but discontinued service integration models can yield important insights into the development, refinement, and sustainability of integration models and strategies.

Reported availability of substance use-related screening and referral varied by geographical region and by income level of the country in which the clinic was located. These services were least commonly reported at clinics within low-income countries and most commonly reported at clinics located within high-income countries. This distribution may reflect the context, including the prevalence of substance use among PLWH as well as the overall (i.e., not only HIV-related) lack of available services and staff trained to screen and treat substance use disorders and other mental health conditions, which is more prominent in resource-restricted countries [47]. Barriers to referral were not assessed in the current study. For some HIV treatment centers, non-referral to substance use treatment is likely informed by a lack of nearby substance use treatment services. In other instances, non-referral may represent a lack of linkage or integration to available substance use treatment services. Future research is needed to understand barriers to referral to substance use treatment at HIV treatment centers across high- and low-resource settings. Implementation research is also needed to identify, implement, and evaluate promising substance use and HIV service integration models, particularly in low-resource, high HIV burden settings [48].

Implementation research to integrate substance use treatment within HIV care settings should consider the context-specific burden of substance use as well as context-specific variability in substances used due to the need for specific treatment interventions for different substances. Medications, for example, are an important component of treatment for opioid use disorder and alcohol use disorder and have been successfully implemented in U.S.-based HIV clinics [32, 49, 50]. Behavioral interventions for substance use disorders have also been successfully implemented with PLWH and at HIV clinics. A meta-analysis of behavioral interventions targeting alcohol use among PLWH (many of which were implemented in HIV clinics) found that such interventions reduced alcohol consumption and improved HIV-related outcomes [51]. Evidence also supports the effectiveness of Screening, Brief Intervention, and Referral to Treatment (SBIRT) approaches to reduce alcohol use in primary and other clinical care settings. Evidence for the effectiveness of SBIRT approaches to reduce other drug use in HIV care settings remains limited. Additional research is needed to identify implementation factors which influence integration of behavioral and medication-based interventions into HIV care settings in low- and high-resource settings.

Several promising strategies for integrating substance use and HIV care in the U.S. have been evaluated and could be adapted for resource-limited settings. Two systematic reviews have focused on studies that evaluated integrated substance use and HIV care [30, 52]. The majority of studies that examined the integration of substance use services into HIV care were conducted in the U.S. and focused on integrating buprenorphine/naloxone into HIV care [30, 52]. Four studies identified in these reviews focused on integrating services to reduce alcohol use into HIV care [53–56]. All interventions were associated with reduced alcohol use. Given that alcohol is the most commonly used substance globally, more attention is needed to understand how to effectively integrate interventions to reduce alcohol use into HIV care [57]. A recent study demonstrated benefit of a stepped care model (involving addiction psychiatry, psychologists, and referral) on drinking and HIV-related outcomes when integrated into HIV clinics to address alcohol use disorder among PLWH [58]. Future work is needed to determine how such a model of care might be adapted to HIV care in low- or middle-income settings. Low-, medium-, and high-resource countries likely need different mental health service components, as a single model is unlikely to fit all settings [59]. Task-shifting or task-sharing approaches that address resource limitations of HIV treatment centers in low-resource settings should be implemented and evaluated. While limited, evidence suggests that alcohol interventions that involve task-shifting can be effective and cost-effective in low-resource settings [56, 60].

Substance use screening and referral were less commonly reported at HIV programs that treat only children or adolescents as compared to programs that treat only adults. This is consistent with data from adolescents and young adults in the general population. In the U.S., it has been estimated that fewer than half of pediatricians screen adolescents for substance use or substance use disorders and less than 10% of adolescents with a substance use disorder are referred to substance use treatment [61–63]. Compared to adults, adolescents are substantially less likely to be offered or engaged in substance use treatment [61, 62]. Data on substance use screening and treatment among adolescents in resource-limited settings are particularly limited. The integration of substance use education, screening and referral into pediatric and adolescent HIV programs is critical as evidence suggests that substance use is commonly reported among adolescents living with HIV, increases during adolescence, and has been associated with suboptimal ART adherence [64–67]. In addition, early initiation of substance use has been associated with HIV risk behaviors and subsequent substance use disorders into adulthood. Additional research is needed to understand how to effectively and sustainably integrate substance use screening and treatment into pediatric HIV treatment settings in resource-constrained settings [68].

This study has several key limitations. Data were collected from HIV treatment sites participating in the IeDEA consortium and may not be representative of all HIV treatment centers in a particular country or region. Because IeDEA sites have the capacity to routinely contribute electronic data, these sites may function at a higher level than sites in that country that are not participating in IeDEA. As such, integration of substance use-related services may be more common at IeDEA sites as compared to HIV treatment centers that are not participating in IeDEA. Information on clinic setting (i.e., urban or rural) was missing for 28 sites and information on level of care (i.e., district hospital, regional hospital) was missing for 27 sites in the 2017 survey. This analysis relied on reports from health facility staff regarding substance use-related services at participating clinics. Availability of reported services was not independently verified, and specific approaches to substance use-related services or services related to individual substances were not assessed. Wide variability likely exists in the quality and procedures related to substance use-related services in HIV clinics in IeDEA.

## Conclusions

This study reports the availability of substance use-related education, screening, and referrals in HIV treatment centers across global settings. Gaps persist particularly in relation to the integration of substance use screening and referral into HIV care settings, especially for pediatric populations and those receiving care within low-income countries. Future research should assess how screening is conducted, for which substances screening is conducted, the use of validated screening tools, how referral or treatment is conducted in response to positive screening, and the quality and consistency of such procedures. Potentially modifiable, multilevel barriers and facilitators to sustainable integration should be identified and promising implementation strategies which address these barriers and facilitators should be developed, implemented, and evaluated. Longitudinal research into the sustainability of various integration models is needed.

## Supporting information

S1 File IeDEA 2014 site survey. (PDF)

S2 File IeDEA 2017 site assessment survey. (PDF)
