# Supplementary material for: Substance use service availability in HIV treatment programs: Data from the global IeDEA consortium, 2014-2015 and 2017
Source: PLoS One. 2020 Aug 27;15(8):e0237772. doi: 10.1371/journal.pone.0237772 (PMC7451518; doi:10.1371/journal.pone.0237772)
Supplement: S1 File — (PDF) [file pone.0237772.s001.pdf]

IeDEA 2014 Site Survey

Record ID ______________________

Please enter the full name of this clinical site (no abbreviations) ______________________
(optional)

Name of person completing this survey ______________________

Email address of the person completing the survey ______________________

Please enter the date this survey is being completed ______________________

Role of the person completing this survey
- ◯ Principal Investigator
- ◯ Regional Data Manager
- ◯ Site Manager
- ◯ Site Data Manager
- ◯ Head Clinician
- ◯ Head Clinical Officer
- ◯ Head Nurse
- ◯ Other

(Pick the response that best describes your role.)

Please specify other role ______________________

Training of the person completing this survey
- ◯ Pediatrician
- ◯ Non-Pediatrician Physician / Consultant / Physician Faculty
- ◯ Medical Officer
- ◯ Clinical Officer
- ◯ Nurse
- ◯ Pharmacist
- ◯ Counselor
- ◯ Data Manager
- ◯ Research Staff
- ◯ Epidemiologist
- ◯ Infectious Disease Specialist
  Other

(Pick the response that best describes your training.)

Please specify other training ______________________

SITE INFORMATION

What is the location of this site?
- ◯ Urban
- ◯ Mostly Urban
- ◯ Mostly Rural
- ◯ Rural
- ◯ Unknown

Is this site a public or private facility?
- ◯ Public
- ◯ Private

Is this site affiliated with an academic institution?
- ◯ Yes
- ◯ No

PATIENT POPULATION

What types of patients are seen at this site?

- ◯ Adults only (ADULT)
- ◯ Children only (PED)
- ◯ Both adults and children (BOTH)

If PED: By what age are pediatric patients expected to move to an adult clinic for care?

____
(years (integer))

If ADULT: What is the minimum age at which patients are enrolled for care?

____
(years (integer))

STAFFING

How often are the following categories of staff available at this site?

	Available Every Day the Clinic is Open	Available Some Days	Never Available
a. Pediatrician (general)	◯	◯	◯
b. Internist, family practitioner, generalist (physician)	◯	◯	◯
c. Mid-level providers (clinical officers, nurse practitioners, physicians assistants)	◯	◯	◯

PREVENTION

Where are these HIV prevention services provided to your patients?

	Provided in this Clinic	In the same Health Facility (but not at this clinic)	Only offsite (at distance)	Not available
a. HIV counseling and testing	◯	◯	◯	◯
b. Counseling regarding disclosure to sexual partners	◯	◯	◯	◯
c. Screening for Sexually Transmitted Infections (STIs)	◯	◯	◯	◯
d. Education on sexual behavior changes and safer sex methods	◯	◯	◯	◯
e. Family planning counseling	◯	◯	◯	◯
f. Provision of condoms	◯	◯	◯	◯
g. Provision of birth control interventions (other than condoms)	◯	◯	◯	◯
h. Services for the prevention of mother-to-child HIV transmission (PMTCT)	◯	◯	◯	◯

	Provided in this Clinic	In the same Health Facility (but not at this clinic)	Only offsite (at distance)	Not available
i. Education on high-risk substance-use behaviors and harm reduction practices	○	○	○	○
j. Screening for drug and alcohol use/abuse	○	○	○	○
k. Referral for substance abuse treatment	○	○	○	○
l. Pre-exposure prophylaxis (PrEP)	○	○	○	○
m. Post-exposure prophylaxi (PEP)	○	○	○	○

If this site provides HIV counseling and testing: What other kinds of HIV testing services does this site offer?

- ☐ Partner testing (sex partners)
- ☐ Testing of family and other household members
- ☐ Home testing kits
- ☐ Testing at locations in the community
- ☐ Other

(Check all that apply)

Other (please specify) ______________________________

If this site provides family planning services: What types?

- ☐ Barrier methods (condoms, diaphragms, cervical cap)
- ☐ Oral contraceptive pills
- ☐ Intrauterine device
- ☐ Sterilization (female)
- ☐ Sterilization (male)
- ☐ Injectable Depo-Provera
- ☐ Other Injectable hormones
- ☐ Birth control sponges
- ☐ Contraceptive patch
- ☐ Vaginal ring
- ☐ Other

(Check all that apply)

Other (please specify) ______________________________

PREVENTION: PMTCT

If this site provides PMTCT services:

Who prescribes ART for women receiving PMTCT services?

- ☐ Nurses
- ☐ Mid-level providers (clinical officers, nurse practitioners, physicians assistants)
- ☐ Physicians/medical officers
- ☐ Other

(Check all that apply)

Other (please specify) ______________________________

What is the predominant WHO option for PMTCT currently offered at this site? (see picture accompanying survey)

- ◯ Option A
- ◯ Option B
- ◯ Option B+

Table 1. Three options for PMTCT programmes

	Woman receives:		
	**Treatment (for CD4 count ≤350 cells/mm³)**	**Prophylaxis (for CD4 count >350 cells/mm³)**	**Infant receives:**
***Option A*[a]**	Triple ARVs starting as soon as diagnosed, *continued for life*	*Antepartum*: AZT starting as early as 14 weeks gestation<br>*Intrapartum*: at onset of labour, sdNVP and first dose of AZT/3TC<br>*Postpartum*: daily AZT/3TC through 7 days postpartum	Daily NVP from birth through 1 week beyond complete cessation of breastfeeding; or, if not breastfeeding or if mother is on treatment, through age 4–6 weeks
***Option B*[a]**	*Same initial ARVs for both*[b]*:*		Daily NVP or AZT from birth through age 4–6 weeks regardless of infant feeding method
	Triple ARVs starting as soon as diagnosed, *continued for life*	Triple ARVs starting as early as 14 weeks gestation and *continued intrapartum and through childbirth if not breastfeeding or until 1 week after cessation of all breastfeeding*	
Option B+	*Same for treatment and prophylaxis*[b]*:*		Daily NVP or AZT from birth through age 4–6 weeks regardless of infant feeding method
	Regardless of CD4 count, triple ARVs starting as soon as diagnosed,[c] *continued for life*		

Note: "Triple ARVs" refers to the use of one of the recommended 3-drug fully suppressive treatment options.

[a] Recommended in WHO 2010 PMTCT guidelines

[b] True only for EFV-based first-line ART; NVP-based ART not recommended for prophylaxis (CD4 >350)

[c] Formal recommendations for Option B+ have not been made, but presumably ART would start at diagnosis.

CLINICAL AND LAB SERVICES

What general clinical services are provided at this site?

- ☐ Blood pressure (BP) monitoring (Hypertension screening)
- ☐ Diabetic screening (hemoglobin A1C testing, oral glucose tolerance testing, fasting glucose, etc.)
- ☐ Height measurement (for purposes of obtaining body mass index (BMI) measurements
- ☐ Treatment of Opportunistic Infections (OIs)
- ☐ Co-trimoxazole for OI prophylaxis (Bactrim, Septra, TMP-SMX)
- ☐ Nutritional supplementation
- ☐ Cervical cancer screening (Pap smear, Visual Inspection with Acetic Acid, etc.)

(Check all that apply)

Do all patients receive co-trimoxazole for OI prophylaxis? (universal therapy for pre-ART and ART patients)

- ◯ Yes
- ◯ No

What types of patients receive co-trimoxazole for OI prophylaxis?

- ☐ Pre-ART patients
- ☐ Patients who meet CD4 or clinical staging criteria
- ☐ Tuberculosis (TB) patients
- ☐ HIV-exposed children
- ☐ HIV-infected children
- ☐ Other

(check all that apply)

Other (please specify) ______________________________

If this clinic sees pediatric patients: What specialized pediatric clinical services are provided at this site?

- ☐ Post-natal ARV prophylaxis
- ☐ Infant feeding counseling
- ☐ Male circumcision for infants
- ☐ Immunizations
- ☐ Nutritional support
- ☐ Growth monitoring
- ☐ Integrated Management of Childhood Illness (IMCI)

(Check all that apply)

Where are these HIV clinical and lab services provided to your patients?

	Provided in this Clinic	In the same Health Facility (but not at this clinic)	Only offsite (at distance)	Not available
a. Sexually transmitted infections (STIs) treatment	○	○	○	○
b. Hepatitis B testing	○	○	○	○
c. Hepatitis C testing	○	○	○	○
d. Hepatitis C treatment	○	○	○	○
e. Tuberculosis (TB) screening/diagnosis (AFB smear microscopy)	○	○	○	○
f. TB screening/diagnosis (Chest X-ray)	○	○	○	○
g. TB diagnosis (culture)	○	○	○	○
h. TB diagnosis (GeneXpert)	○	○	○	○
i. TB treatment (provision of anti-tuberculous therapy)	○	○	○	○
j. TB prevention (administration of Isoniazid preventative therapy-IPT)	○	○	○	○

ART ADHERENCE

How is ART medication adherence monitored in patients on ART at this site?

- ☐ Number of missed follow-up visits, including clinical and medication pick up visits
- ☐ Viral load (when applicable)
- ☐ Patient self-recall (asking patient about completed/missed doses the past 3d, 7d, and 1 month)
- ☐ Pharmacy pickup
- ☐ Medication tracking (pill counts)
- ☐ Other

(Check all that apply)

Other (please specify) ______________________________

What ART adherence support services are offered at this site?

- ☐ Cell phone/SMS reminders
- ☐ One-on-one counseling
- ☐ Group counseling
- ☐ Patient education media (written, pictorial, video, etc.)
- ☐ Pill boxes or blister packs
- ☐ Calendars, checklists, or other reminders
- ☐ Alarm clocks, wrist watches, beepers
- ☐ Pharmacist included on multidisciplinary team
- ☐ Routine review of medication pick up
- ☐ Other

(Check all that apply)

Other (please specify) ______________________________

OUTREACH

Does this site have a system to track patients who miss appointments?

- ○ Yes
- ○ No

What is done if a patient misses an appointment?

- ☐ Phone call to individual
- ☐ Phone call to family
- ☐ Send letter
- ☐ Send SMS
- ☐ Send email
- ☐ Home visit by clinic staff
- ☐ Home visit by community outreach worker
- ☐ Other

(Check all that apply)

Other (please specify) ______________________________

How do you track patients who are lost to followup at this site?

- ☐ Review of hospital records
- ☐ Consult with pharmacy
- ☐ Review national death registry
- ☐ Check other national system (insurance, HIV drug pickup registry, etc.)
- ☐ Wait for notification from a family member/friend
- ☐ Determined during outreach visits
- ☐ Other

(Check all that apply)

Other (please specify) __________________________________

PHARMACY

What medications are dispensed at this site?

- ☐ HIV antiretroviral medications (ARVs)
- ☐ Isoniazid
- ☐ TB medications other than isoniazid
- ☐ Malaria treatment
- ☐ Fluconazole
- ☐ Amphotericin B
- ☐ Pegylated interferon and ribavirin
- ☐ Hepatitis C protease inhibitors (telaprevir, boceprevir, etc.)

(Check all that apply)

Who dispenses most of the ARVs at this site?

- ◯ Pharmacists
- ◯ Pharmacy assistants (technicians)
- ◯ Nurses
- ◯ Other

Other (please specify) __________________________________

In the past 12 months, has this site had medication drug supply disruptions/stockouts lasting 1 week or longer?

- ◯ Yes
- ◯ No
- ◯ Don't know

If Yes -- what medications had supply interruptions/disruptions in the past 12 months?

- ☐ HIV antiretroviral medications (ARVs)
- ☐ Co-trimoxazole (Bactrim, Septra, TMP-SMX)
- ☐ Isoniazid
- ☐ TB medications other than isoniazid
- ☐ Fluconazole
- ☐ Other

(Check all that apply)

Other (please specify) __________________________________

In the past 12 months, has this site had patients on a waiting list to receive ART?

- ◯ Yes
- ◯ No
- ◯ Don't Know

NUTRITION

What nutritional services are provided at this site?

- ☐ Nutritional assessment (body measurements and dietary assessment)
- ☐ Nutritional counseling
- ☐ Micronutrients/vitamin supplements
- ☐ Food supplements
- ☐ None of the above

(Check all that apply)

LABORATORY

Is CD4+ cell count testing used to monitor immunologic status of HIV+ patients at this site?

- ◯ Yes, routinely
- ◯ Yes, but not routinely
- ◯ No, not available

Where is the laboratory that conducts the majority of the CD4 cell count testing for this site?

- ◯ Onsite, at the same health facility as the HIV clinic
- ◯ Offsite, at a distance

Is Viral Load testing used to monitor status of HIV+ patients at this site?

◯ Yes, routinely
◯ Yes, but not routinely
◯ No, not available

Where is the laboratory that conducts the majority of the Viral Load testing for this site?

◯ Onsite, at the same health facility as the HIV clinic
◯ Offsite, at a distance

What other types of labs are available for routine patient care at your site?

☐ Hemoglobin
☐ Creatinine
☐ Serum Cholesterol
☐ Triglycerides (blood lipid panels)
☐ AST (SGOT) and/or ALT (SGPT)
☐ Syphilis screening/testing (RPR)
☐ Early infant diagnosis (DNA or RNA PCR)
☐ Cryptococcal meningitis screening (serum cryptococcal antigen)
☐ Cryptococcal meningitis screening (using the lateral flow assay (using specimens from the blood, urine, and/or cerebrospinal fluid (CSF))
☐ Cryptococcal meningitis diagnosis (CSF India Ink and/or CSF cryptococcal antigen)
☐ HIV-1 genotypic drug resistance testing
☐ TB drug resistance testing
☐ Rapid HIV tests
(Check all that apply)

TUBERCULOSIS

If this site provides TB screening, diagnosis, and/or treatment.: Who is the best point of contact for TB information at your site?

◯ Me
◯ Someone else

Name of person (if not provided previously) __________________________________

Email of person (if not provided previously) __________________________________

CANCER

Does this site screen, diagnose, or treat any type of cancer?

◯ Yes
◯ No

Who is the best point of contact for cancer information at your site?

◯ Me
◯ Same person as TB contact, if applicable
◯ Someone else

Name of person (if not provided previously) __________________________________

Email of person (if not provided previously) __________________________________

FEEDBACK

You have reached the end of the IeDEA 2014 Site Survey. Thank you for taking the time to complete it.

Do you have any comments on the survey or recommendations for future surveys? __________________________________
