# Supplementary material for: Substance use service availability in HIV treatment programs: Data from the global IeDEA consortium, 2014-2015 and 2017
Source: PLoS One. 2020 Aug 27;15(8):e0237772. doi: 10.1371/journal.pone.0237772 (PMC7451518; doi:10.1371/journal.pone.0237772)
Supplement: S2 File — (PDF) [file pone.0237772.s002.pdf]

IeDEA 2017 Site Assessment Survey

The purpose of this survey is to learn about the clinical and support services provided to HIV patients who are enrolled in care at this health facility. This survey is being conducted at all health facilities participating in the International Epidemiology Databases to Evaluate AIDS (IeDEA) network.

This survey is intended to be completed by staff who have in-depth knowledge about the care and services provided to adult HIV patients in the HIV clinic or within the health facility or institution. Most questions refer to care and services provided within the HIV clinic. If your health facility does not have a dedicated clinic for HIV care and treatment, please answer for the facility overall, regardless of what unit(s) serves these patients. If your health facility has multiple HIV care and treatment clinics that serve different patient groups, please report on the services provided for adult HIV patients, unless otherwise indicated.

A few questions in this survey may require consultation with staff in specific units, such as laboratory, pharmacy, HIV counseling and testing units, etc.

There are no incorrect answers to this survey. The information you provide is important for understanding how health facility and service delivery characteristics relate to patient outcomes of interest.

Thank you for your time completing this survey. We are very grateful for your participation.

QUESTIONS	RESPONSE FIELDS
1. RESPONDENT INFORMATION	
1.1 Name of person completing this survey	
1.2. Email address of the person completing the survey	
1.3. Please enter the date this survey is being completed	__ __/__ __/ 201__<br>(DD / MM / YYYY)
1.4. What is your title?	☐ Head Clinician/Clinical Officer In-Charge<br>☐ Other clinician<br>☐ Site Manager<br>☐ Site Data Manager<br>☐ Head Nurse<br>☐ Other (specify) ____________________
2. HEALTH FACILITY INFORMATION	
2.1. What is the location of this health facility?	☐ Urban<br>☐ Mostly urban<br>☐ Mostly rural<br>☐ Rural<br>☐ Unknown
2.2. What type of facility (i.e. level of care) is this site?	☐ Primary (health center)<br>☐ District hospital<br>☐ Regional, provincial or teaching hospital
2.3. Is this site a public or private facility?	☐ Public<br>☐ Private
2.4. Is this health facility affiliated with an academic institution?	☐ Yes<br>☐ No
3. PATIENT POPULATION	
3.1 What types of HIV patients are seen at this health facility's HIV Care and Treatment (C&T) clinic(s)?<br>*Select one response only*	☐ Adults only {→ SKIP to 4.1}<br>☐ Children/adolescents only {→ SKIP to 3.2}<br>☐ Adults and children/adolescents
3.1a. At this health facility, are HIV patients of all ages (adults, children, adolescents) served within the same Care and Treatment (C&T) clinic or are specialized clinics held for different age groups?	☐ Yes (all patients seen within single clinic) {→ SKIP to 4.1}<br>☐ No (dedicated clinics held for different patient groups)

QUESTIONS	RESPONSE FIELDS		
3.1b. How often are specialized or dedicated HIV Care and Treatment (C&T) clinics held for each of the following patient groups?	Available every day the health facility is open	Available on special/ dedicated days	Never available
a. Adult patients	☐	☐	☐
b. Pediatric patients	☐	☐	☐
c. Adolescent/young people (ages 10-24)	☐	☐	☐
3.2. By what age are pediatric or adolescent patients expected to transition to an adult clinic for care?	☐ ____________(years)		
4. STAFFING & COMMUNITY LINKAGES			
4.1. How often are the following categories of staff available at this C&T clinic?	Available every day clinic is open	Available some days	Never available
a. Pediatrician (general)	☐	☐	☐
b. Internist, family practitioner, generalist (physician)	☐	☐	☐
c. Mid-level providers (clinical officers, nurses/nurse practitioners, midwives, physician assistants)	☐	☐	☐
d. Adherence counselors	☐	☐	☐
e. Peer educators/mentors/navigators	☐	☐	☐
f. Outreach workers	☐	☐	☐
g. Nutritionists	☐	☐	☐
4.2. Does this C&T clinic routinely work with any of the following community-based organizations to raise awareness about HIV/AIDS, promote HIV testing, or trace patients?<br><br>*Check all that apply OR select "None/Not applicable."*	☐ None/Not applicable<br>☐ Community health committees or village health teams<br>☐ Community health workers and/or volunteers<br>☐ Voluntary and/or community-based organizations (CBOs)<br>☐ People living with HIV/AIDS (PLWHA) associations or support groups<br>☐ Community leaders/officials<br>☐ Youth groups or peer educator groups<br>☐ Other (specify)______________________________		
5. HIV TESTING			
5.1. How often are HIV counseling and testing services offered at this health facility?	☐ Services available every day clinic is open<br>☐ Services available some days<br>☐ Services never available **{→ SKIP TO 5.2}**		
5.1a. What types of HIV testing services are offered at this health facility?<br><br>*Check all that apply.*	☐ Opt-out testing (provider-initiated)<br>☐ Opt-in testing (client-initiated)<br>☐ Partner/couples testing<br>☐ "Family tree" testing (testing of family and other household members)<br>☐ Rapid HIV tests/Same-day testing<br>☐ HIV self-testing kits		
5.1b. What type of support services are routinely provided to clients who receive an HIV+ test result at this health facility?<br><br>*Check all that apply OR select "None". **Routinely provided** means provided as the standard of care.*	☐ None<br>☐ Psychosocial support from nurse or social worker<br>☐ Psychosocial support from lay counselor, peer educator, mentor<br>☐ Partner disclosure counseling and support<br>☐ Referral to support groups<br>☐ Referral to community-based volunteers/workers<br>☐ Other (specify)______________________________________		
5.2. Which of the following types of HIV testing services are regularly available <u>in the catchment area</u> of this health facility?<br><br>*Check all that apply OR select "None". **Regularly available** means that these testing services are offered frequently or on a predictable schedule (weekly, monthly, etc.)*	☐ None<br>☐ Home testing<br>☐ Voluntary counseling and testing (VCT) at fixed community locations<br>☐ Mobile VCT testing<br>☐ Other (specify)______________________________________		

QUESTIONS	RESPONSE FIELDS
**6. ENTRY/ENROLLMENT INTO CARE**	
6.1. What are the most common entry points into HIV care for patients at this health facility?<br>*Check all that apply.*	☐ Voluntary counseling and testing (VCT) unit<br>☐ Antenatal care (ANC) unit or prevention of mother to child transmission (PMTCT)<br>☐ Labor and delivery unit<br>☐ Sexually transmitted infection (STI) treatment unit<br>☐ Tuberculosis (TB) unit<br>☐ Outpatient department<br>☐ Referrals from other health facilities/sites<br>☐ Other (specify) ______________________________
6.2. After a positive result on an HIV screening test at this health facility or elsewhere, is additional testing done to confirm the diagnosis of HIV infection for patients enrolling into care at this site?	☐ Yes<br>☐ No **{→ SKIP TO 6.3}**
6.2a. How is the diagnosis of HIV infection confirmed for patients enrolling into care at this site?<br>*Check all that apply.*	☐ Confirmatory antibody test<br>☐ Confirmation based on CD4 count<br>☐ Confirmation based on HIV viral load (PCR) test<br>☐ Other (specify)______________________
6.3. Are any incentives provided for patients to enroll in HIV care early (e.g., within one month of diagnosis)?	☐ Yes<br>☐ No **{→ SKIP TO 7.1}**
6.3a. What type of incentives are provided for patients to enroll in HIV care early?<br>*Check all that apply.*	☐ Mobile airtime voucher<br>☐ Cash transfers for transportation costs<br>☐ Other (specify) _____________________________
**7. PRE-ART PATIENT RETENTION AND OUTREACH STRATEGIES**	
7.1. Do you provide services/care to any **pre-ART patients** at this C&T clinic?	☐ Yes<br>☐ No **{→ SKIP TO 8.1}**
7.2 What medications are routinely provided to **pre-ART patients** (or routinely prescribed if your clinic does not provide medications directly)?<br>*Check all that apply OR select "None".*<br>***Routinely provided** means provided (or prescribed) as the standard of care.*	☐ None<br>☐ Cotrimoxazole<br>☐ Isoniazid<br>☐ Vitamin supplements (i.e., multivitamins)<br>☐ Other (specify) _________________________
7.3. Which of the following commodities/supplies are routinely provided to **pre-ART patients**?<br>*Check all that apply OR select "None".*<br>***Routinely provided** means provided as the standard of care.*	☐ None<br>☐ Vitamin supplements<br>☐ Food supplements<br>☐ Condoms<br>☐ Soap<br>☐ Pill boxes<br>☐ Appointment calendar<br>☐ Patient education materials<br>☐ Other (specify)_______________
7.4 What is done if **pre-ART patients** miss an appointment?<br>*Check all that apply OR select "Nothing".*	☐ Nothing *(No routine follow-up tracing of pre-ART patients)*<br>☐ Phone call to individual<br>☐ Phone call to family<br>☐ Send letter<br>☐ Send SMS<br>☐ Send email<br>☐ Send message via online patient portal<br>☐ Home visit by clinic staff<br>☐ Home visit by community outreach worker<br>☐ Other (specify) ________________

QUESTIONS	RESPONSE FIELDS	
**8. ART INITIATION**		
8.1 What are the current criteria for ART initiation at this health facility and when were those criteria introduced?<br>*Check all that apply, and specify the year that the guideline was introduced at this facility.*<br>*As relevant, specify the types of patients for whom the treatment guidance is followed—e.g., pregnant patients, TB patients, pediatric, etc.*	TREATMENT CRITERIA	MONTH & YEAR introduced at facility (MM/YYYY)
	☐ Start **all** patients on ART regardless of CD4 or symptoms	__ __/ 20__ __
	☐ Start **some** patients on ART regardless of CD4 or symptoms (specify)______________________________	__ __/ 20__ __
	☐ CD4 count ≤500 cells/mm³ for all patients	__ __/ 20__ __
	☐ CD4 count ≤500 cells/mm³ for some patients (specify)____________________________________	__ __/ 20__ __
	☐ CD4 count ≤350 cells/mm³ for all patients	__ __/ 20__ __
	☐ CD4 count ≤350 cells/mm³ for some patients (specify)____________________________________	__ __/ 20__ __
	☐ Other (specify)_________________________________	__ __/ 20__ __
8.2 On average, at this clinic, how soon after establishing treatment eligibility do patients initiate ART?<br>*Check one best response.*	☐ Same day that ART eligibility is established<br>☐ 1-14 days after establishing ART eligibility<br>☐ 2-4 weeks after establishing ART eligibility<br>☐ >1 month after establishing ART eligibility	
8.3 At this clinic how many ART readiness counseling sessions are typically conducted before eligible patients initiate ART?<br>*Check one best response.*	☐ 0 sessions<br>☐ 1 session<br>☐ 2 sessions<br>☐ 3 sessions<br>☐ 4 or more sessions	
**9. ART ADHERENCE AND RETENTION STRATEGIES**		
9.1 How is ART medication adherence routinely monitored in patients on ART at this C&T clinic?<br>*Check all that apply OR select "Not applicable".*<br>*Routinely monitored means monitored as the standard of care.*	☐ Not applicable *(Medication adherence not routinely monitored)*<br>☐ Unstructured assessment of adherence by clinician<br>☐ Structured assessment of adherence by clinician using recall instrument (e.g., recall of missed doses during 24-hour, 3-day, 7-day, 30-day, or other recall period).<br>☐ Pill counts<br>☐ Pharmacy refills<br>☐ Electronic dose monitoring (MEMS caps)<br>☐ Directly observed treatment<br>☐ Routine viral loads<br>☐ Viral loads for patients suspected of non-adherence.<br>☐ Other (specify)________________________________	
9.2 What is the standard frequency of refills for patients who are stable on ART?<br>*Check one best response.*	☐ Monthly<br>☐ Every 3 months<br>☐ Every 6 months<br>☐ Other (specify)____	
9.3 What ART adherence support services are routinely provided to HIV patients at this C&T clinic?<br>*Check all that apply OR select "None/Not applicable".*<br>*Routinely provided means provided as the standard of care.*	☐ None/Not applicable<br>☐ One-on-one adherence counseling<br>☐ Group adherence counseling<br>☐ Individual mental health counseling<br>☐ Group mental health counseling<br>☐ Referral to peer support or mentor groups<br>☐ Other (specify) ____	
9.4. Which of the following types of adherence aids/reminders are routinely provided to ART patients?<br>*Check all that apply. Routinely provided means provided as the standard of care.*	☐ None/Not applicable<br>☐ Patient education media (written, pictorial, video, etc.)<br>☐ Pill boxes or blister packs<br>☐ Calendars, checklists, or other reminders<br>☐ Alarm clocks, wrist watches, beepers<br>☐ Pharmacist included on multidisciplinary team<br>☐ Routine review of medication pick up<br>☐ Other (specify) ____	

QUESTIONS	RESPONSE FIELDS
9.5. Which of the following commodities and adherence supports are routinely provided to ART patients?<br>*Check all that apply OR select "None/Not applicable".*<br>**Routinely provided** *means provided as the standard of care.*	☐ None/Not applicable<br>☐ Vitamin supplements<br>☐ Food supplements<br>☐ Assistance with transport costs<br>☐ Condoms<br>☐ Soap<br>☐ Appointment calendar<br>☐ Other (specify)______________________________
9.6. Which of the following types of cell phone or SMS reminders are used with ART patients at this C&T clinic?<br>*Check all that apply OR select "None/Not applicable".*	☐ None/Not applicable **{→ SKIP TO** 9.7}<br>☐ Voice messaging<br>☐ Text messaging<br>☐ Interactive text messaging<br>☐ Interactive voice messaging<br>☐ Other (specify) ______________________________
9.6a. How are SMS/phone reminders used to support ART adherence and retention in care?<br>*Check all that apply.*	☐ Reminders to take medication<br>☐ Reminders of upcoming appointments<br>☐ Follow-up to missed appointments<br>☐ General health education (HIV and other diseases)<br>☐ Other (specify) ____________________
9.7. What is done to track ART patients who miss appointments?<br>*Check all that apply, OR select "Nothing/No follow-up".*	☐ Nothing/No follow-up with ART patients who miss appointments<br>☐ Phone call to individual<br>☐ Phone call to family<br>☐ Send letter<br>☐ Send SMS<br>☐ Send email<br>☐ Send message via online patient portal<br>☐ Home visit by clinic staff<br>☐ Home visit by community outreach worker<br>☐ Other (specify) ______________________________

10. PREVENTION SERVICES FOR HIV POSITIVE PATIENTS					
10.1. Which of the following prevention services are provided to **enrolled HIV patients** and where are these services provided?	Provided in C&T Clinic	In same health facility (but not at C&T clinic)	Only offsite (distance ≤30 minutes travel)	Only offsite (distance > 30 minutes travel)	Not available
a. Counseling regarding disclosure to sexual partners	☐	☐	☐	☐	☐
b. Screening for Sexually Transmitted Infections (STIs)	☐	☐	☐	☐	☐
c. Education on sexual behavior changes and safer sex methods	☐	☐	☐	☐	☐
d. Condoms	☐	☐	☐	☐	☐
e. Family planning counseling	☐	☐	☐	☐	☐
f. Family planning/contraceptive methods other than condoms	☐	☐	☐	☐	☐
g. Education on high-risk substance-use behaviors and harm reduction practices	☐	☐	☐	☐	☐
h. Screening for drug and alcohol use/abuse	☐	☐	☐	☐	☐
i. Referral for substance abuse treatment	☐	☐	☐	☐	☐
j. Pre-exposure prophylaxis (PrEP) for serodiscordant couples	☐	☐	☐	☐	☐
k. Post-exposure prophylaxis (PEP) for serodiscordant couples	☐	☐	☐	☐	☐
l. Voluntary male circumcision services for serodiscordant couples.	☐	☐	☐	☐	☐
m. HPV vaccine					
n. Cervical cancer screening (e.g., visual inspection with acetic acid/PAP smear)	☐	☐	☐	☐	☐
o. Anal PAP screening	☐	☐	☐	☐	☐

QUESTIONS	RESPONSE FIELDS
10.2. What is the predominant WHO option for Prevention of Mother to Child Transmission (PMTCT) currently offered at this health facility?	☐ Option A (for CD4≦350: ARVs starting at diagnosis and continued for life; for CD4>350: ARVs starting at or after 14 weeks gestation and continuing through 7 days postpartum).<br>☐ Option B (For CD4≦350: ARVs starting at diagnosis and continued for life; for CD4> 350: ARVs starting at or after 14 weeks gestation and continued through childbirth or 1 week after cessation of breastfeeding)<br>☐ Option B+ (For any CD4 count: ARVs starting at diagnosis and continued for life).<br>☐ None (Do not provide PMTCT services)
**11. OTHER SERVICES PROVIDED TO HIV PATIENTS**	
11.1. Which of the following services are provided to **pediatric HIV patients** at this health facility?<br><br>*Check all that apply or select "Not Applicable" if no pediatric patients are served at this health facility.*	☐ Not applicable *(No pediatric patients served at this health facility)*<br>☐ Postnatal ARV prophylaxis<br>☐ ART initiation<br>☐ Infant feeding counseling<br>☐ Male circumcision for infants<br>☐ Immunizations<br>☐ Nutritional support<br>☐ Growth monitoring<br>☐ Integrated Management of Childhood Illness (IMCI)
11.2. Does this health facility offer any of the following services for adolescent/youth HIV patients?<br><br>*Check all that apply or tick "None".*	☐ None *(No dedicated services for adolescent patients)*<br>☐ Dedicated hours or space for youth/adolescent HIV testing & counseling services<br>☐ Dedicated hours or space for youth/adolescent HIV care and treatment services<br>☐ Peer counseling for youth/adolescent HIV patients<br>☐ Support groups specifically for youth/adolescent HIV patients

11.3. Which of the following services are provided to **enrolled HIV patients** and where are these services provided?	Provided in C&T Clinic	In same health facility (but not at C&T clinic)	Only offsite (distance ≤30 minutes travel)	Only offsite (distance > 30 minutes travel)	Not available
a. Prenatal care (also known as antenatal care)	☐	☐	☐	☐	☐
b. Maternity care (labor/delivery)	☐	☐	☐	☐	☐
c. Postpartum care	☐	☐	☐	☐	☐
d. TB treatment	☐	☐	☐	☐	☐
e. Where do HIV patients who become pregnant get their PMTCT services?	☐	☐	☐	☐	☐

11.4. Do any patients at this C&T clinic receive TB prophylaxis (administration of Isoniazid preventive therapy-IPT)?	☐ Yes<br>☐ No **{→ SKIP TO 11.6}**
11.5. After screening negative for active TB, what types of patients receive TB prophylaxis (administration of Isoniazid preventative therapy-IPT)?<br><br>*Check all that apply.*	☐ Patients who meet CD4 or clinical staging criteria<br>☐ Patients with positive TB skin test or IGRA<br>☐ Patients deemed at risk for TB (e.g., living with family/household members with TB, incarcerated, or homeless)<br>☐ Other (specify) ____________________________
11.6. Do patients other than TB patients and pregnant women at this C&T clinic receive Cotrimoxazole prophylaxis as the standard of care?	☐ Yes<br>☐ No
**12. LABORATORY & DIAGNOSTIC SERVICES**	
12.1. Is CD4+ cell count testing used to monitor immunologic status of <u>pre-ART patients</u> at this health facility?	☐ Yes, routinely (i.e., at regular intervals or milestones)<br>☐ Yes, but not routinely<br>☐ No, not available
12.2. Is CD4+ cell count testing used to monitor immunologic status of <u>ART patients</u> at this health facility?	☐ Yes, routinely (i.e., at regular intervals or milestones)<br>☐ Yes, but not routinely<br>☐ No, not available
12.3. Where is the laboratory that conducts the majority of the CD4 cell count testing for this C&T clinic?	☐ Onsite, at the same health facility as the HIV clinic<br>☐ Offsite, ≤ 30 minutes travel time<br>☐ Offsite, at a distance (>30 minutes travel time)
12.4 Are same-day/point of care (POC) CD4 count results routinely available at this health facility?<br><br>**Routinely available** means that the test could be requested or performed today, if needed.	☐ Yes<br>☐ No

QUESTIONS	RESPONSE FIELDS
12.4a. During the past month, what was the usual turnaround time *(in hours OR days)* for getting CD4 count results?<br>***Turnaround time** means the time from ordering or referring a patient for the test to the time when results are received.*	☐ ___________hours<br>☐ ___________days
12.5. Is viral load testing used to monitor status of HIV+ patients at this health facility?	☐ Yes, routinely *(i.e., at regular intervals or milestones)*<br>☐ Yes, but not routinely<br>☐ No, not available **{→ SKIP TO 1**2.6}
12.5a. Where is the laboratory that conducts the majority of viral load testing for this C&T clinic?	☐ Onsite, at the same health facility as the HIV clinic<br>☐ Offsite, ≤ 30 minutes travel time<br>☐ Offsite, at a distance (>30 minutes travel time)
12.5b. During the past month, what was the usual turnaround time (in days) for getting viral load test results?<br>***Turnaround time** means the time from ordering or referring a patient for the test to the time when results are received.*	☐ ___________days

12.6 Which of the following tests are available for patients at this C&T clinic?<br>*Confirm the availability of each test with laboratory staff involved in screening and diagnostic testing. "**Available**", means that the test could be requested or performed today, if needed, and you are confident about receiving results*	Test/results available for routine patient care	Test/results not available for routine patient care	Test available but no longer in routine use
a. 1st generation rapid diagnostic immunoassay tests (Western Blot, HIV-1 LFA)	☐	☐	☐
b. 2nd generation rapid diagnostic immunoassay (late antibody (IgG) only)	☐	☐	☐
c. 3rd generation rapid diagnostic immunoassay (late & early (IgG/IgM) antibody (Ab))	☐	☐	☐
d. 4th generation rapid immunoassay (IgG/IgM antibody and antigen (Ag/Ab))	☐	☐	☐
e. 4th generation chemiluminescent immunoassay (Ag/Ab)	☐	☐	☐
f. HIV 1/2 immunoassay (supplemental or confirmatory HIV 1/2 laboratory-based diagnostic)	☐	☐	☐
g. P24 ELISA (laboratory-based immunoassay (Ag))	☐	☐	☐
h. Qualitative PCR	☐	☐	☐
i. Quantitative PCR or viral load assay	☐	☐	☐
j. DNA or RNA PCR for early infant diagnosis (EID)	☐	☐	☐
k. HIV-1 genotypic drug resistance testing	☐	☐	☐

12.7. What other types of labs and diagnostic services are available for routine care of HIV patients at your C&T clinic?<br>*Confirm the availability of each test with laboratory staff involved in screening and diagnostic testing. "**Available for routine care**", means that the test could be requested or performed today, if needed, and you are confident about receiving results*	Test/results available for routine patient care	Test/results not available for routine patient care
a. Hemoglobin	☐	☐
b. Creatinine	☐	☐
c. Serum Cholesterol	☐	☐
d. Triglycerides	☐	☐
e. AST (SGOT) and/or ALT (SGPT)	☐	☐
f. Syphilis screening/testing (RPR)	☐	☐
g. Cryptococcal meningitis screening (serum cryptococcal antigen)	☐	☐
h. Cryptococcal meningitis screening using the lateral flow assay (using specimens from the blood, urine, and/or cerebrospinal fluid (CSF))	☐	☐
i. Cryptococcal meningitis diagnosis by CSF India Ink	☐	☐
j. Cryptococcal meningitis diagnosis by CSF latex agglutination	☐	☐
k. TB screening/diagnosis (AFB smear microscopy)	☐	☐
l. TB screening/diagnosis (Chest X-ray)	☐	☐
m. TB diagnosis (culture)	☐	☐
n. TB diagnosis (GeneXpert)	☐	☐
o. TB drug resistance testing	☐	☐

QUESTIONS	RESPONSE FIELDS
13. PHARMACY	
13.1. Is there a pharmacy located at this health facility?	☐ Yes<br>☐ No **{→ SKIP TO 1**4.1}
13.2. Which of the following medications are dispensed and/or available at this health facility?<br>*Check all that apply.*	☐ First-line HIV antiretroviral medications (ARVs)<br>☐ Second-line HIV ARVs<br>☐ Isoniazid<br>☐ TB medications other than isoniazid<br>☐ Cotrimoxazole (Bactrim, Septra, TMP-SMX)<br>☐ Malaria treatment<br>☐ Fluconazole<br>☐ Amphotericin B<br>☐ Hepatitis C Direct Acting Antivirals (DAAs) — e.g., telaprevir, bocepravir, sofosbuvir/ledipasvir
13.3. In the past 12 months, has this health facility had medication drug supply disruptions/stock outs lasting 1 week or longer?	☐ Yes<br>☐ No **{→ SKIP TO 1**3.5}<br>☐ Don't know **{→ SKIP TO 1**3.5}
13.4. What medications had supply disruptions/stock outs in the past 12 months?<br>*Check all that apply.*	☐ First-line HIV antiretroviral medications (ARVs)<br>☐ Second-line HIV ARVs<br>☐ Isoniazid<br>☐ TB medications other than isoniazid<br>☐ Cotrimoxazole (Bactrim, Septra, TMP-SMX)<br>☐ Malaria treatment<br>☐ Fluconazole<br>☐ Amphotericin B<br>☐ Hepatitis C Direct Acting Antivirals (DAAs) — e.g., telaprevir, bocepravir, sofosbuvir/ledipasvir
13.5. In the past 12 months, has this C&T clinic had patients on a waiting list to receive ART?	☐ Yes<br>☐ No<br>☐ Don't know
14. MEDICAL RECORDS AND PATIENT TRACKING	
14.1. Do you track the outcomes of patients who are lost to follow-up (e.g. outcomes such as patient deaths, transfers to other facilities, ART status, etc.)?	☐ Yes<br>☐ No **{→ SKIP TO** END}
14.2 Are the outcomes of tracked patients recorded in electronic databases?	☐ Yes<br>☐ No **{→ SKIP TO** END}
14.3. After tracking patients lost to follow-up at this C&T clinic, what information is recorded in electronic databases?<br>*Check all that apply.*	☐ Transfers to other facilities<br>☐ Loss to follow-up<br>☐ Deaths<br>☐ Other (specify) ___________________
15. ACKNOWLEDGEMENTS (OPTIONAL)	
15.1. We would like to acknowledge clinic team members who participated in the completion of this survey. If your team members would like their names included, please enter their full names, separated by commas, so we can acknowledge their contribution.	

1-MAY-17

Thank you for your participation.
